# Cervical Spine Fracture Prediction by Simple Plain X‐Ray in Ankylosing Spondylitis Patients after Low‐Energy Trauma

**DOI:** 10.1111/os.13423

**Published:** 2022-09-30

**Authors:** Bingchuan Liu, Yitian Gao, Kaifeng Ye, Zhongwei Yang, Guojin Hou, Zhishan Zhang, Hongquan Ji, Fang Zhou, Yun Tian

**Affiliations:** ^1^ Department of Orthopedics Peking University Third Hospital Beijing China; ^2^ Beijing Key Laboratory of Spinal Disease Research Peking University Third Hospital Beijing China

**Keywords:** Ankylosing spondylitis, Cervical spine fracture, Emergency treatment, X‐ray

## Abstract

**Objective:**

Timely diagnosis is essential in the management of cervical spine fracture (CSF) in ankylosing spondylitis (AS) patients. However, the value of simple plain X‐ray in the early management of ASCSF has not been well‐studied. This study aimed to explore the prediction ability of simple plain X‐ray for CSF in AS patients who suffer from low‐energy trauma (LET).

**Methods:**

From January 2010 to December 2020, AS patients who experienced LET were retrospectively reviewed. Clinical data including gender, age, body mass index, time interval between AS diagnosis and trauma, smoking or not, and a presence of continuous bony bridge between anterior margin of C1 and C2 body or not were collected. Morphological features including atlanto‐occipital gap, Pavlov ratio of C2–7, Angle A–D, Borden's index, and Harrison's value were measured by the lateral cervical X‐ray. All data was compared between patients who had CSF and those who did not. Binary logistic regression analysis and receiver operator characteristic (ROC) curves were applied to discriminate and assess the predictive parameters.

**Results:**

A total of 129 AS patients were divided into Fracture group (41 cases) and Non‐fracture group (88 cases) based on whether CSF existed. Twelve parameters showed significant differences between two groups (*p* < 0.05). According to the binary logistic regression model, four of the 12 parameters showed a further correlation with the occurrence of CSF, namely, mean Pavlov ratio (*p* < 0.001, OR = 0.067, 95% CI: 0.023 to 0.194), Angle D (*p* = 0.031, OR = 1.057, 95% CI: 1.005 to 1.112), Borden's index (*p* = 0.042, OR = 1.131, 95% CI: 0.994 to 1.287), the time interval between the AS diagnosis and the trauma (*p* < 0.020, OR = 0.935, 95% CI: 0.883 to 0.990). The ROC curve further revealed the mean Pavlov ratio had the largest AUC (0.793) with the cut‐off of 0.72. While the optimal cut‐off value was 45.65° for Angle D (sensitivity = 61.0%, specificity = 78.4%), 9.79 for Borden's index (sensitivity = 87.8%, specificity = 37.5%), 15.50 years for the time interval between AS diagnosis and trauma (sensitivity = 70.7%, specificity = 56.8%).

**Conclusions:**

The time interval between the AS diagnosis and the trauma, mean Pavlov ratio, Angle D, and Borden's index showed predictive ability for the occurrence of CSF in AS patients who encounter LET. Surgeons should consider measuring these parameters in the management of AS patient.

## Graphical abstract

Patients with ankylosing spondylitis (AS) have a significantly higher risk of cervical spine fracture (CSF) after injury than normal people. However, hidden CSF can easily be missed when the patient experiences minor trauma or light symptoms. By measuring X‐ray parameters, we initially found indicators that could predict the risk of CSF in AS patients suffering low‐energy trauma. This innovative discovery was conducive to detect CSF early, thereby reducing the missed diagnosis rate and improving medical safety.

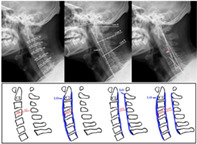

## Introduction

Ankylosing spondylitis (AS) is a chronic inflammatory disease which predominantly affects the axial skeleton, causing ossification of paraspinal ligaments and intervertebral discs, leading to decreased spinal flexibility, damaged bone structure, and impaired balance control.^1^, ^2^, ^3^ These adverse factors jointly lead to high incidence of post‐traumatic spine facture in AS patients, even after a low‐energy trauma (LET).^4^, ^5^ Accounting for up to 78% of all AS spinal fractures,^6^ cervical spine is most prone to fracture in AS patients. Timely diagnosis is an essential prerequisite for both prompt, effective intervention and favorable prognosis in the therapeutic strategy of cervical spine fracture (CSF) with AS (ASCSF).

X‐ray is usually deemed as a primary option to discriminate the potential fracture in patients suffering from a trauma under emergency conditions, but revealing the fracture configuration is not sufficient to ASCSF diagnosis and is difficult for interpretation by non‐expert doctors. Simple two‐dimensional X‐ray images cannot clearly display anatomical structures and minor fracture lines, given the highly abnormal spinal structure in patients with AS, including ossified ligaments, surrounding osseous proliferation, poor outlining of the disc space, and osteoporosis.^7^, ^8^ Moreover, the diagnosis may be complicated by the presence of long‐standing pain and the application of corticosteroid therapy and NSAIDs, which can mask the acute fracture symptoms.^9^ In a study by Caron *et al*., 30% of the AS cases with spine fractures did not initially obtain correct diagnosis.^10^ Additionally, Anwar *et al*.^7^ found that the diagnosis was missed in up to 59.4% of the cases when conventional radiography was used. Unfortunately, such a delay could result in kyphosis worsening and an increase in the risk of neurological complications. In clinical practice, although computed tomography (CT) and magnetic resonance imaging (MRI) can help to detect obscure fractures that are not visible on plain radiographs,^11^, ^12^ these techniques are costly and require longer appointment intervals, and thus these advanced examinations might not be readily available to patients suffering from LET. Therefore, the risk of missed diagnosis would be decreased and the service efficiency would be improved if the risk of ASCSF could be predicted *via* simple X‐ray radiography, followed by CT or MRI, reasonably recommended based on the predicted results.

In the present study, we attempted to predict the risk of ASCSF in patients suffering from LET from daily life by measuring certain morphological features. To our knowledge, no existed reports have mentioned such an exploration. The aims of this study were (i) to compare morphologic parameters in AS patients who underwent LET with CSF and those without, (ii) to investigate predictive parameters for the occurrence of CSF in AS patients who encounter LET, and (iii) to determine the cutoff values of parameters with predictive ability. Our hypothesis was that reasonable radiographical predictors could effectively increase potential for early diagnosis of CSF in AS patients suffering from trivial trauma and symptoms and would further contribute to the reduction of the occurrence of disastrous complications due to delayed or missed diagnosis.

## Materials and Methods

### Study Design

From January 2010 to December 2020, AS patients who experienced LET such as simple falls from standing or sitting height were initially recruited in this retrospective study. The following inclusion criteria were applied: (1) age over 18; (2) good mental health; and (3) complete post‐traumatic CT or MRI that confirmed the existence of cervical fracture. The exclusion criteria were as follows: (1) high‐energy and severe trauma such as from traffic or high‐fall injury; (2) incomplete clinical materials. The clinical and radiological data of the patients were acquired by reviewing their medical history and measuring the values by the Picture Archiving and Communication Systems (PACS). Basic data were collected by reviewing patients' medical records, including gender, age, body mass index (BMI), time interval between AS diagnosis and trauma, smoking or not, and a presence of continuous bony bridge between anterior margin of C1 and C2 body or not. The study was reviewed and approved by the Ethics Committee of Peking University Third Hospital, Beijing, China (No. M2017331).

### Radiological Measurement

Radiological data were obtained by lateral cervical X‐ray which was performed on patients in the neutral position. All data were evaluated using a radiography information system (Centricity RIS‐IC CE V3.0; GE Healthcare, Little Chalfont, UK). The following parameters were measured.

#### Atlanto‐Occipital Gap

Atlanto‐occipital gap (X1) represents the distance from atlas to occiput, indicating the neck extension of AS patients. On the lateral cervical X‐ray in the neutral position, the line connecting the upper margin of posterior tubercle of atlas and the lower border of occiput was drawn and the distance was defined as Atlanto‐occipital gap.

#### Pavlov Ratio

**Fig. 1:**
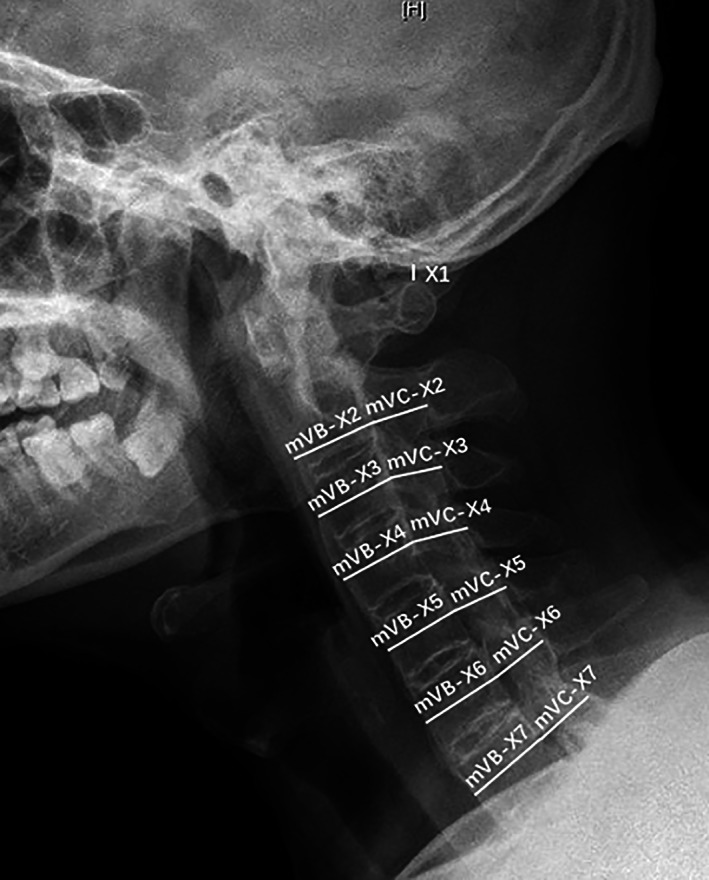
Distance measurement on the lateral cervical X‐ray in the neutral position. X1, atlanto‐occipital gap; mVB‐X(2–7), modified sagittal diameter of C2 to C7 body; mVC‐X(2–7), modified sagittal diameter of C2 to C7 canal (VB, vertebral body; VC, vertebral canal)

**Fig. 2:**
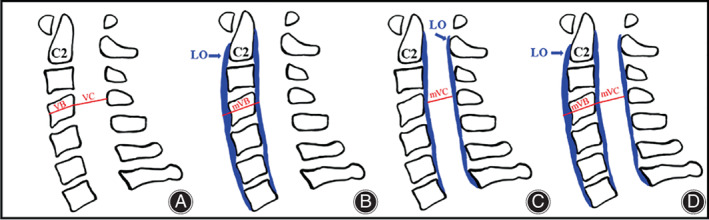
The illustration of the measurement difference between normal and modified VB and VC. (A) Normal sagittal diameter of VB and VC; (B) sagittal diameter of mVB (the blue arrow indicates the ossification of paraspinal ligaments); (C) sagittal diameter of mVC; (D) sagittal diameter of mVB and mVC (VB, sagittal diameter of vertebral body; VC, sagittal diameter of vertebral canal; LO, ligament ossification; mVB, modified sagittal diameter of vertebral body; mVC, modified sagittal diameter of vertebral canal)

Pavlov ratio is the quotient of vertebral sagittal diameter of the cervical spinal canal divided by sagittal diameter of the vertebral body, which is used to evaluate spinal canal stenosis. In this study, Pavlov ratio was measured and calculated according to a modified method from Aebli *et al*.^13^ As displayed in Figure 2, mVB was the sum of the thickness of the anterior longitudinal ligament ossification, vertebral body diameter, and the thickness of the posterior longitudinal ligament ossification, while mVC indicated the distance between the posterior longitudinal ligament ossification and the yellow ligament ossification. On the lateral cervical X‐ray in the neutral position (Figure 1), mVB was measured from the anterior border of the anterior longitudinal ligament ossification to the posterior border of posterior longitudinal ligament ossification. Similarly, mVC was measured from the mid points of the posterior border of posterior longitudinal ligament ossification to the ossification of yellow ligaments ossification. mVC/mVB was expressed as a Pavlov ratio.

#### Intersection Angles

**Fig. 3:**
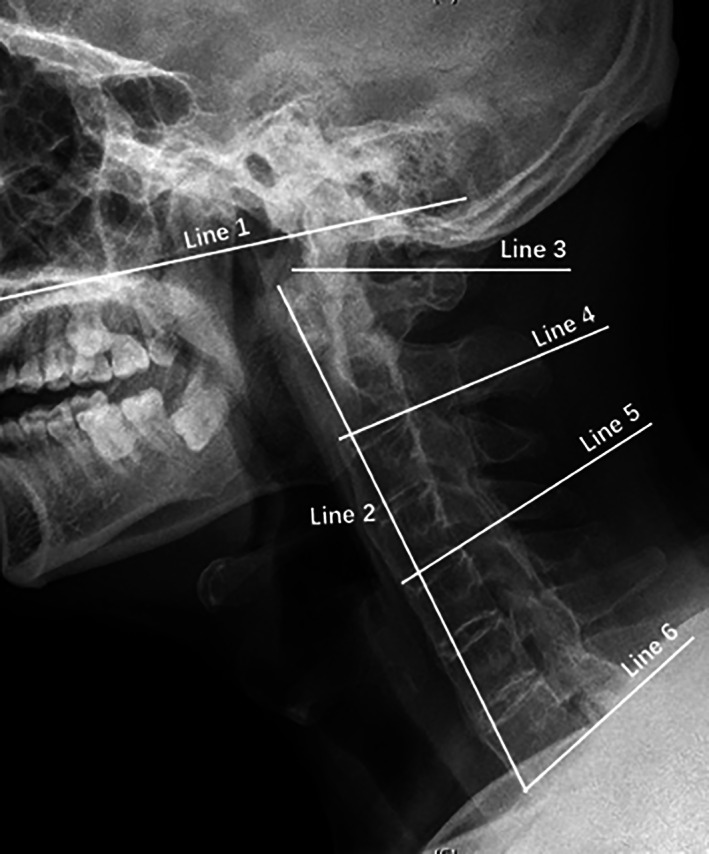
Angle measurement on the lateral cervical X‐ray in the neutral position. Line 1, a line parallel to hard palate; Line 2, a line passing through anterior‐inferior border of the sixth cervical vertebra and the most anterior aspect of the first cervical vertebra; Line 3, a line parallel to the upper border of C1 body; Line 4, a line passing through the anterior‐inferior point and the posterior‐inferior point of C2 body; Line 5, a line passing through the anterior‐inferior point and the posterior‐inferior point of C4 body; Line 6, a line passing through the anterior‐inferior point and the posterior‐inferior point of C7 body

Certain intersection angles were also measured to evaluate range of motion of the cervical spine (Figure 3). Firstly, Line 1 was drawn paralleling to the hard palate and Line 2 was drawn passing through anterior‐inferior border of the sixth cervical vertebra and the most anterior aspect of the first cervical vertebra. The intersection angle between Line 1 and Line 2 was identified as Angle A. Secondly, Line 3 was drawn parallelly to the upper border of C1 body. Lines 4–6 were consequently drawn passing through the anterior‐inferior point and the posterior‐inferior point of C2, C4, and C7 body. Angle B indicated the intersection angle between Line 3 and Line 4; Angle C showed the intersection angle between Line 3 and Line 5; Angle D was used for the intersection angle between Line 3 and Line 6.

#### Borden's Index and Harrison's Value

**Fig. 4:**
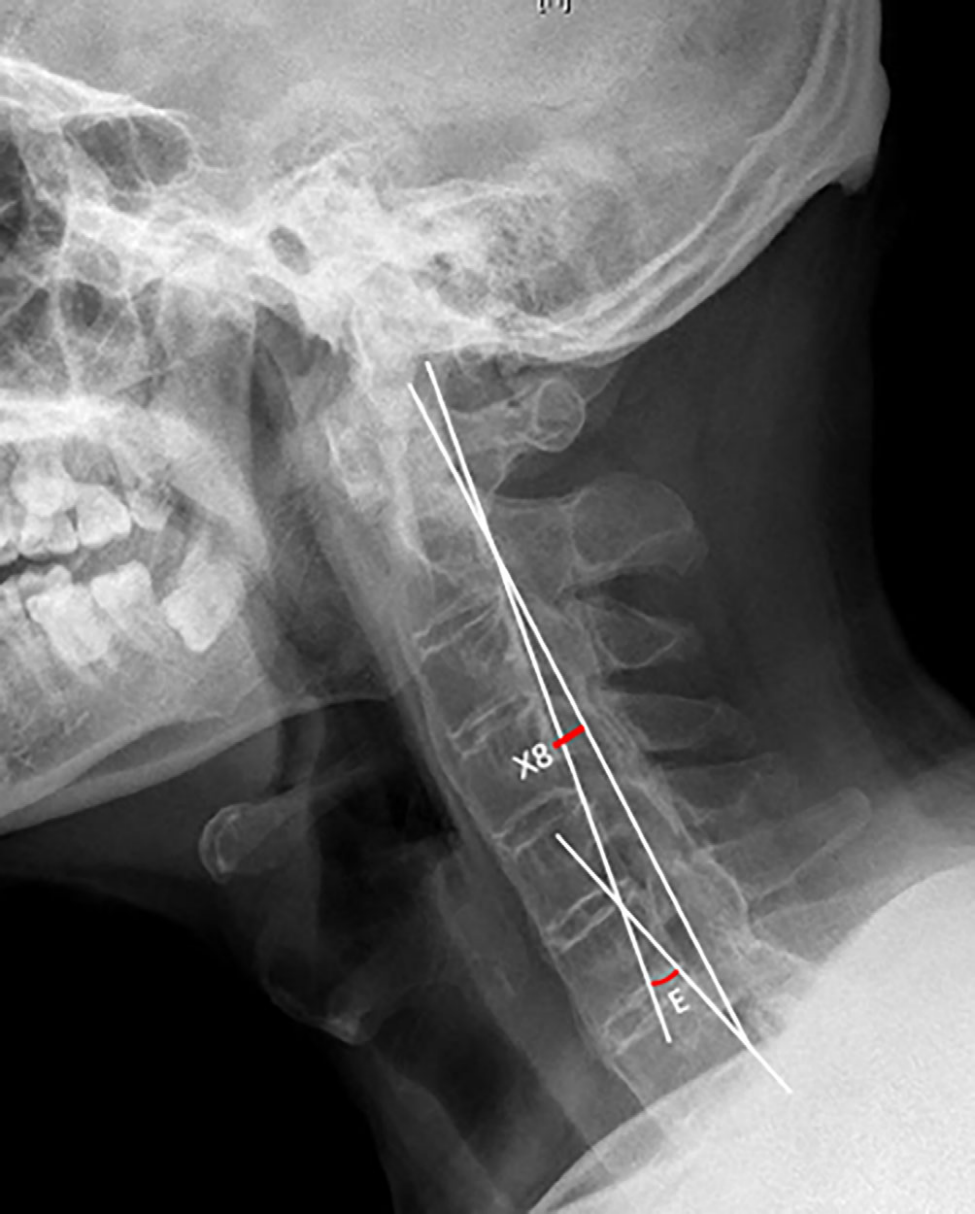
Measurement of cervical curvature by Borden's index and Harrison's value on the lateral cervical X‐ray in the neutral position. X8, the vertical distance from the midpoint of C4 posterior marginal to the line passing through the posterior superior marginal of C2 odontoid process and posterior inferior edge of C7 body; Angle E, the intersection angle between tangent lines along the cervical curve of the posterior margins of C2 and C7 body

Borden's index (X8)^14^ and Harrison's value (Angle E)^15^ were further applied to reflect the cervical curvature (Figure 4). The line passing through the posterior superior marginal of C2 odontoid process and posterior inferior edge of C7 body was drawn and its vertical distance to the midpoint of C4 posterior marginal was identified as Borden's index (X8). To acquire Harrison's value (Angle E), two lines parallel to the posterior margins of C2 and C7 body was drawn along the cervical curve. The intersection angle between these two lines (Angle E) was identified as Harrison's value.

To determine the interobserver reliability, all radiographic measurements were performed by two blinded authors independently on a sample of 60 patients randomly selected (20 patients from fracture group and 40 patients from non‐fracture group). Additionally, one author repeated all measurements on the same group of patients 2 weeks later to access intraobserver reliability.

### Statistical Analysis

SPSS 22.0 software was used to conduct statistical analysis. The normality of the distribution was determined by Kolmogorov–Smirnov test. Normally distributed continuous variables were analyzed by independent‐sample *t‐*test, whereas non‐normal variables were assessed by Mann–Whitney test. Categorical data were evaluated by the chi‐square test. Then, a binary logistic regression model was applied to discriminate among multivariate predictors. Odds ratio (OR) and 95% confidence interval (95% CI) revealed the strength of each association. The receiver operating characteristic (ROC) curve was employed to describe the discrimination ability of the predictive indicators. Area under the curve (AUC) was applied as a quantitative index. Youden's index (=sensitivity + specificity − 1) was calculated, and the highest score was considered as an optimal predictive cut‐off value. *p* < 0.05 was considered to indicate statistically significant differences. The interobserver and intraobserver reliability were determined by the intraclass correlation (ICC). An ICC value greater than 0.9 was considered excellent and a value between 0.75 and 0.9 was considered good. An ICC value between 0.5 and 0.75 was considered moderate and a value less than 0.5 was deemed poor.

## Results

### Patents' Characteristic and Radiographic Measurements

A total number of 129 patients (mean age = 52.4 ± 8.1 years) were enrolled in this study, including 119 males and 10 females. The patients were divided into a Fracture group (41 cases) and a Non‐fracture group (88 cases) based on the existence of a cervical fracture. Patients' demographics and measurement data were listed in Table 1. According to the statistical analysis results, 12 parameters had significant differences between the two groups (*p* < 0.05), namely, the time interval between the AS diagnosis and the trauma (*p* = 0.013), the presence of a continuous bony bridge between the anterior margin of the C1 and C2 body (*p* < 0.001), Pavlov ratio of C2 to C7, mean Pavlov ratio (*p* < 0.001), Angle D (*p* = 0.001), Borden's index (*p* = 0.013), and Harrison's value (*p* = 0.001).

**TABLE 1:** Demographics and measurement data between the two groups

Parameters	Fracture group (*n* = 41)	Non‐fracture group (*n* = 88)	Statistic (*χ* ^2^/*z*/*t*)	*p*‐Value
Gender (*n*)			0.694	0.41
Male	39 (95.1%)	80 (90.9%)		
Female	2	8		
Age (years)	54.1 ± 11.7	51.5 ± 10.2	1.288	0.200
BMI (kg/m^2^)	23.8 ± 4.4	24.9 ± 3.6	−1.546	0.125
Time interval between AS diagnosis and trauma (years)	21.5 ± 11.1	16.6 ± 7.3	−2.490	0.013
Smoke (*n*)			2.023	0.155
Yes	12 (29.3%)	16 (18.2%)		
No	29	72		
Presence of continuous bony bridge between C1 and C2			14.355	<0.001
Yes	18 (43.9%)	12 (13.6%)		
No	23	76		
X1 (mm)	5.9 ± 2.5	5.3 ± 3.3	0.890	0.375
Pavlov ratio				
C2	0.71 ± 0.16	0.79 ± 0.12	−2.842	0.004
C3	0.63 ± 0.13	0.75 ± 0.13	−4.987	<0.001
C4	0.61 ± 0.14	0.76 ± 0.15	−5.385	<0.001
C5	0.64 ± 0.13	0.78 ± 0.16	−4.980	<0.001
C6	0.67 ± 0.14	0.80 ± 0.15	−4.488	<0.001
C7	0.66 ± 0.11	0.80 ± 0.14	−5.099	<0.001
Mean Pavlov ratio	0.65 ± 0.10	0.77 ± 0.14	−5.355	<0.001
Angle A (°)	101.84 ± 7.62	101.23 ± 10.09	0.348	0.729
Angle B (°)	37.97 ± 7.38	35.33 ± 8.61	1.692	0.093
Angle C (°)	44.20 ± 8.11	44.01 ± 11.09	0.099	0.921
Angle D (°)	45.13 ± 9.39	52.61 ± 11.98	−3.460	0.001
Borden's index (mm)	5.87 ± 3.00	8.28 ± 4.94	−2.486	0.013
Harrison's value (°)	15.61 ± 8.14	22.68 ± 12.01	−3.222	0.001

The time interval between the AS diagnosis and the trauma was much longer for patients in fracture group when compared with the non‐fracture group (*p* = 0.013). However, no difference was reported for smoking between two groups.

Bony bridge between the anterior margin of the C1 and C2 body was also more commonly observed in patients who had CSF than those who did not (*p* < 0.001). Mean Pavlov ratio was found to be significantly decreased in the fracture group compared with the non‐fracture group. Similar findings were also reported for all Pavlov ratios of C2 to C7 (all *p* < 0.05). The patients in the fracture group had significantly smaller Angle D (*p* = 0.001) with respect to the control group. Also, a lower Borden's index (*p* = 0.013) and Harrison's value (*p* = 0.001) prevailed in Fracture group when compared with the Non‐fracture group. However, no difference in radiological measurements was reported for Atlanto‐occipital gap (X1) and Angle A to Angle C between the two groups.

### Reliability Assessment

Interobserver and intraobserver ICCs of all radiographic measurements performed was presented in Table 2 along with their 95% CIs. A good to excellent intraobserver reliability was determined by the intraobserver ICCs ranging from 0.82 to 0.96. The interobserver ICCs ranged from 0.75 to 0.90, indicating a good interobserver reliability.

**TABLE 2:** Interobserver and intraobserver reliability of radiographic measurements performed*

Parameters	Interobserver reliability	Intraobserver reliability
X1	0.90 (0.85–0.93)	0.90 (0.85–0.93)
Pavlov ratio		
C2	0.84 (0.76–0.89)	0.95 (0.92–0.96)
C3	0.82 (0.74–0.88)	0.94 (0.92–0.96)
C4	0.86 (0.79–0.90)	0.96 (0.94–0.97)
C5	0.83 (0.75–0.89)	0.95 (0.93–0.97)
C6	0.86 (0.79–0.90)	0.96 (0.93–0.97)
C7	0.87 (0.82–0.91)	0.95 (0.92–0.96)
Angle A	0.81 (0.73–0.87)	0.83 (0.75–0.88)
Angle B	0.82 (0.73–0.87)	0.85 (0.78–0.90)
Angle C	0.83 (0.75–0.89)	0.82 (0.74–0.88)
Angle D	0.85 (0.78,0.90)	0.86 (0.79–0.90)
Borden's index	0.90 (0.86–0.93)	0.88 (0.83–0.92)
Harrison's value	0.75 (0.65–0.83)	0.80 (0.72–0.87)

* Values are presented as ICC along with their %95 CIs.

### Predictive Risk Factors of CSF

Based on the binary logistic regression model (Forward: LR) presented in Table 3, four of the 12 mentioned parameters with significant differences between two groups showed a further correlation with the occurrence of cervical fracture after LET in AS patients. These included the mean Pavlov ratio (*p* < 0.001, OR = 0.067, 95% CI: 0.023 to 0.194), Angle D (*p* = 0.031, OR = 1.057, 95% CI: 1.005 to 1.112), Borden's index (*p* = 0.042, OR = 1.131, 95% CI: 0.994 to 1.287), the time interval between the AS diagnosis and the trauma (*p* < 0.020, OR = 0.935, 95% CI: 0.883 to 0.990).

**TABLE 3:** The binary logistic regression model (Forward: LR) of the enrolled variables

Parameters	B	Wald	*p*‐Value	OR	95% CI
Mean Pavlov ratio	−2.704	24.867	<0.001	0.067	0.023, 0.194
Angle D	0.056	4.648	0.031	1.057	1.005, 1.112
Borden's index	0.123	3.482	0.042	1.131	0.994, 1.287
Time interval*	−0.068	5.370	0.020	0.935	0.883, 0.990

* *Time interval* indicates time interval between AS diagnosis and trauma.

### Cutoff Values of PTS and ATT for Predicting CSF

**Fig. 5:**
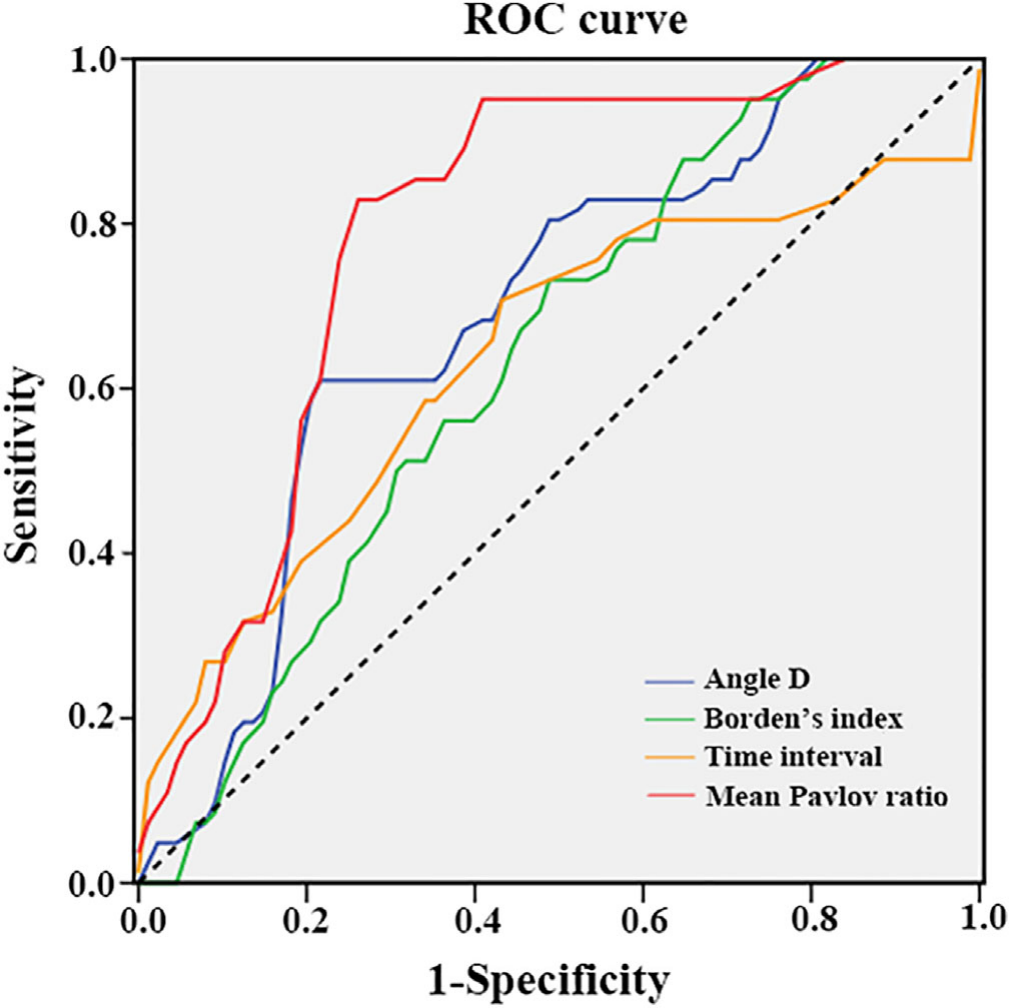
ROC curve of the four parameters including Angle D, Borden's index, Time interval (time interval between AS diagnosis and trauma), and mean Pavlov ratio

The ROC curve and the AUC were used to further understand the predictive ability of the four parameters established by the logistic regression model. As presented in Table 4 and Figure 5, the highest AUC was obtained for the mean Pavlov ratio (0.793, 95% CI: 0.873 to 0.986), which was fairly effective for predicting an increased risk of CSF in AS patients who underwent LET considering the optimal cutoff value of 0.72 (sensitivity = 0.829, specificity = 0.739). The AUCs of Angle D, Borden's index, and the time interval between the AS diagnosis and the trauma ranged between 0.6 and 0.7.

**TABLE 4:** The AUC and the optimal cut‐off value based on the highest Youden's index

Parameters	AUC	Highest Youden's index	Optimal cut‐off value	Sensitivity	Specificity
Mean Pavlov ratio	0.793	0.568	0.72	0.829	0.739
Angle D	0.690	0.394	45.65	0.610	0.784
Borden's index	0.636	0.253	9.79	0.878	0.375
Time interval*	0.636	0.275	15.50	0.707	0.568

* *Time interval* indicates time interval between AS diagnosis and trauma.

## Discussion

The principal findings of this study can be summarized as follows: (1) AS patients who had CSF *vs* patients who did not after LET exhibit different morphological features on simple X‐ray; (2) mean Pavlov ratio, Angle D, Borden's index, the time interval between the AS diagnosis and the trauma showed a significant correlation with the occurrence of ASCSF; and (3) mean Pavlov ratio demonstrated the best predictive ability for ASCSF with the cutoff value of 0.72 (sensitivity = 0.829, specificity = 0.739). Our research findings will be crucial to clinical emergency practice, especially considering that ASCSF in most cases was caused by LET according to previous reports.^4^, ^7^

### Decreased Pavlov Ratio

An important reason why patients with AS are susceptible to LET is that the continuous ligament ossification and degenerative discs collectively reduce cervical elasticity, whose mechanical function behaves as a long force arm like extremities, acting as a rigid lever, incapable of appropriately dissipating the energy of a traumatic event.^8^ Besides, a thicker ossification is associated with higher stiffness and fragility; the fused spinal columns have lost their elasticity and movements resulting in altered biomechanics. In this study, to establish the severity of ligament ossification, measurements of the sagittal diameter of both the vertebral body and the canal were accomplished considering the ossification of the anterior longitudinal ligament, posterior longitudinal ligament, and ligamentum flavum. As can be seen in Figure 2, thicker ossification of the anterior and posterior longitudinal ligament caused longer mVB (Figure 2B), whereas thicker ossification of the posterior longitudinal ligament and ligamentum flavum caused shorter mVC (Figure 2C). Additionally, thicker ossification of the three ligaments can cause both shorter mVC and longer mVB (Figure 2D), and these three factors can decrease the Pavlov ratio (=mVC/mVB). According to the statistical results, the Pavlov ratios of C2 to C7 in Fracture group were all significantly lower than that in Non‐fracture group (*p* < 0.05). Furthermore, the results of the binary logistic regression and ROC curve showed that the mean Pavlov ratio was significantly correlated to the occurrence of cervical fracture in AS patients who suffered from LET. The highest AUC of the mean Pavlov ratio manifested its best predictive ability among other parameters. The cut‐off value of the mean Pavlov ratio was 0.72 (sensitivity = 0.829, specificity = 0.739), indicating that for patients with AS who encountered LET, those whose mean Pavlov ratio was less than 0.72 had a higher risk of cervical fracture. From this premise, if the X‐ray examination appears normal, further CT and MRI are highly recommended. In current clinical practice, the Pavlov ratio is usually applied to determine the presence of developmental cervical canal stenosis, which can be diagnosed when the ratio is less than 0.75.^13^ The present study revealed another diagnostic value of Pavlov ratio for the first time and provided an important reference for clinical decision‐making.

### Abnormal Intersection Angles

The maintenance of cervical natural physiological lordosis contributes to buffering the action of a force when the skull and the neck suffer from trauma.^16^, ^17^ The aggravating cervical rigidity caused by chronic inflammation leads to a reduction in the cervical buffer capacity. Besides, stress concentration becomes more pronounced under cervical rigidity, and the mobility trend in the vertical and horizontal directions becomes more obvious, which causes a state of instability of the cervical spine. In the present study, a total number of three parameters related to cervical curvature showed a significant difference between the two groups (*p* < 0.05), namely, Angle D (the intersection angle between the line parallel to the upper border of C1 body and the line passing through the anterior‐inferior point and the posterior‐inferior point of C7 body), Borden's index, and Harrison's value. In addition, straight cervical curvature for all three parameters (namely lesser cervical lordosis) was all exactly detected in the Fracture group. In the further analysis, Angle D and Borden's index were incorporated into the binary logistic regression and ROC curve, and their AUC were 0.690 and 0.636, respectively. The cut‐off value of Angle D and Borden's index were 45.65° and 9.79 mm, indicating a higher possibility of cervical fracture in AS patients suffering from LET when Angle D and Borden's index are lower than 45.65° and 9.79 mm. This finding also suggests that extra care and caution might be needed in examining and treating to avoid iatrogenic trauma especially when a patient's cervical curvature becomes stiff.

### Increased Time Interval between the AS Diagnosis and Trauma

Time interval between AS diagnosis and trauma is an objective reflection index for the severity of AS progression. Theoretically speaking, a longer time interval might be related to more serious ligament ossification and cervical rigidity. Besides, as previously reported, the risk of incurring a spine fracture after injury in AS grows gradually with time, and the risk of sustaining a vertebral fracture could reach an added 1.3% per year.^18^ A study conducted by Deminger *et al*.^19^ explored the spinal radiographic progression in AS based on the modified Stoke Ankylosing Spondylitis Spine Score (mSASSS). They found that the mean progression was 1.6 mSASSS units over 5 years (*p* < 0.001). Other studies revealed the progression of mean 4.2 per 4 years^20^ and 1.3 mSASSS units per year.^21^ An investigation including 132 AS patients in the OASIS (Outcome in AS International Study) cohort further revealed that new syndesmophytes occurred in 33% and 48% of the patients after 2 and 4 years, respectively.^20^ Though the predictive ability of time interval between AS diagnosis and trauma was not high with its AUC of 0.636, the time interval of the Fracture group was significantly longer in the present study (21.5 years *vs* 16.6 years, *p* = 0.013). The cut‐off value of the time interval between the AS diagnosis and the trauma was 15.50 years, indicating that if a patient who encountered LET more than 15.50 years after the diagnosis of AS, they should be considered with increased vigilance to have a potential cervical fracture to avoid missed diagnosis.

### Presence of Continuous Bony Bridge between C1 and C2

In the present study, the morbidity of the continuous bony bridge between C1 and C2 also had significant difference between the two groups (43.9% *vs* 13.6%, *p* < 0.001). The atlantoaxial joint plays an important role in the cervical natural motion, especially in the rotation function. A recent *in vivo* study showed that the flexion‐extension of C1–2 was 13.7 ± 4.2°, accounting for 14.5% of the overall flexion‐extension ROM; the lateral bending neck motion of C1–2 was 7.6 ± 2.7°, accounting for 13.2% of the overall lateral bending ROM; and the axial torsion neck motion of C1–2 was 72.9 ± 7.6°, accounting for 73.2% of the overall rotation ROM.^22^ Previous investigations also achieved similar results.^23^, ^24^, ^25^ Once the motion of C1–2 joint is restricted by the anterior continuous bony bridge, the bearing force capacity from the skull and the neck is in turn reduced, which further increases the risk of cervical fracture.

### Limitation

There are some limitations in our study. On the one hand, the present study included a relatively small number of patients, a larger sample size and a multi‐center study might make the results more convincing. On the other hand, this was a retrospective study, and a prospective study for predicting the possibility of CSF in AS patients suffering minor trauma might have the potential to provide more references to clinical practice.

### Conclusion

In present study, different morphological features were observed between AS patients who had CSF and those who did not. Among these parameters, Angle D, mean Pavlov ratio, Borden's index, and the time interval between the AS diagnosis and the trauma were found to be further correlated with the occurrence of ASCSF and revealed a satisfying predicative ability. This study confirmed the potential of early diagnosis of CSF in AS patients suffering from trivial trauma and symptoms by simple plain X‐ray. More parameters need to be identified in future research. Besides, we call for prospective studies to confirm the predictive factors discovered in our study.

## Author's Contribution

YT and FZ provided the conception and designed this study. BL, YG, KY, ZY contributed to data collection. GH, ZZ, and HJ contributed to data analysis and interpretation. This manuscript was prepared by BL and YG. All authors read and approved the final manuscript.

## Conflict of Interest

The authors of this article declare no conflict of interest.

## Ethics Statement

All authors listed meet the authorship criteria according to the latest guidelines of the International Committee of Medical Journal Editors, and all authors are in agreement with the manuscript.
